# Fundus Lesions in Patients Hospitalized With COVID-19 Infection in Mumbai, India: A Retrospective Review

**DOI:** 10.7759/cureus.11512

**Published:** 2020-11-16

**Authors:** Salil Mehta, Prahlad Prabhudesai

**Affiliations:** 1 Ophthalmology, Lilavati Hospital, Mumbai, IND; 2 Chest Medicine, Lilavati Hospital, Mumbai, IND

**Keywords:** covid-19 mumbai, chorioretinal inflammation, fundoscopy

## Abstract

Background and objective

Coronavirus disease 2019 (COVID-19) is a viral infection that has grown to be a global pandemic, and it is caused by severe acute respiratory syndrome coronavirus 2 (SARS-CoV-2). The ocular involvement in COVID-19, both in the anterior and posterior segments, is increasingly being recognized by ophthalmologists. We report the fundus photographic and systemic findings in 25 patients without recent-onset visual symptoms who were hospitalized with COVID-19.

Methods

Patients with COVID-19 infection who were admitted to an isolation ward/ICU in Mumbai, India during June-August 2020 underwent a comprehensive clinical and systemic evaluation. We performed a fundus evaluation using a handheld fundus camera during their admission period. We conducted a retrospective case record review and extracted demographic characteristics, laboratory findings, and fundus photographs from each case record.

Results

We screened 25 non-consecutive patients, and they included 20 (80%) men and five (20%) women, with ages ranging from 31 to 79 years (mean: 56.3 years). Systemically, the spectrum of severity on admission varied from mild to moderate to severely ill. The majority of the patients had no complaints of recent visual loss. An analysis of fundus photographs of 50 eyes of 25 patients revealed no evidence of fundus lesions in as many as 48 photographs. Two photographs of two eyes of patients showed incidental lesions.

Conclusions

We found no evidence of vascular, inflammatory, or thromboembolic disease that could be linked to COVID-19 infection in any of the images we studied; however, fundus examination may be utilized in patients with co-infection.

## Introduction

Coronavirus disease 2019 (COVID-19) is a highly transmissible viral infection caused by severe acute respiratory syndrome coronavirus 2 (SARS-CoV-2); it has been declared a global health emergency with as many as 47 million cases reported worldwide to date [1]. India has emerged as a hotspot of COVID-19 with an estimated 8.3 million cases and about 123,000 deaths so far [2]. While there is increasing evidence of anterior segment involvement in COVID-19 in the form of congestion or conjunctivitis, posterior segment findings such as hemorrhages or cotton wool spots are still infrequently reported. In this study, we report the fundus photographic and systemic findings in 25 patients hospitalized with COVID-19.

## Materials and methods

Patients who presented with symptoms suggestive of COVID-19 (fever, breathlessness, cough) during the months of June-August 2020 to a tertiary care hospital in Mumbai, India, were triaged with a clinical evaluation and a nasopharyngeal swab for reverse transcription-polymerase chain reaction (RT-PCR) evaluation. Patients with a positive test result were admitted to the COVID-19 ward/ICU as needed. All patients underwent a comprehensive clinical and systemic evaluation. At the discretion of the admitting physician, patients underwent complete and differential blood counts, C-reactive protein (CRP), procalcitonin (PCT), serum D-dimer, serum fibrinogen, serum interleukin-6 (IL-6) assay, and chest X-rays or high-resolution CT (HRCT) scans, or any other investigations deemed relevant. Also, at the discretion of the admitting physician, either as part of an institutional protocol that mandated dilated fundus examination for patients with pyrexia of unknown origin or as part of an evaluation for associated co-infection, a fundus evaluation was performed. Following consent and dilatation, a trained retinal physician used a handheld fundus camera (Zeiss Visuscout 100, Carl Zeiss Meditec, Dublin, CA) to perform bedside bilateral fundus photography. The Visuscout uses a 5-megapixel (MP) camera that permits documentation of the central 40 degrees. The institutional ethics committee granted permission for this study and the relevant information was extracted from patient records.

## Results

Over a period of three months (990 admissions for COVID-19 in this period), we screened 25 non-consecutive patients who were referred by infectious disease consultants in the COVID-19 ward or ICU. These included 20 (80%) men and five (20%) women, with ages ranging from 31 to 79 years (mean: 56.3 years). Systemically, the spectrum of severity on admission varied from uncomplicated illness (three, 12%) to severe pneumonia/acute respiratory distress syndrome (ARDS) (22, 88%) [3]. At the time of evaluation, six (24%) patients were intubated with mechanical ventilation (three females, three males, ages ranging from 46-78 years, mean: 59.6 years) whereas 19 were either only monitored or were on high flow nasal oxygen (HFNO)/non-invasive ventilation (NIV). Six (24%) patients (three females, three males, ages from 46-78 years, mean: 61.5 years) died whereas 19 (two females, 17 males, ages ranging from 31-79 years, mean: 46.36 years) survived and were discharged to home quarantine. Pre-existing comorbidities included seven (28%) patients (seven males, ages from 53-78 years, mean: 62.4 years) with type 2 diabetes mellitus, five (20%) patients (five males, ages from 60-79 years, mean: 70.0 years) with hypertension, and four (16%) patients with chronic kidney disease (CKD) (four males, ages from 57-79 years, mean: 67.25 years). Two (8%) patients presented with a thromboembolic phenomenon: a 54-year-old male with cerebral infarct (frontal, parietal, and occipital lobes), and a 60-year-old male with acute ST-elevation myocardial infarction (STEMI). Three (12%) patients developed thromboembolic phenomenon during this admission. These included a 46-year-old male with bilateral upper limb arterial thrombus, a 51-year-old female with a unilateral thrombosis of the radial artery, and a 43-year-old female with a distal popliteal artery thrombus. One (4%) patient, a 48-year-old male, developed acute bilateral visual loss (counting fingers bilaterally) for which an urgent CT scan of the brain was performed. The results were normal and vision returned to normal within 24 hours, suggesting a transient ischemic attack (TIA). Two (8%) patients (a 46-year-old male and a 56-year-old female) were confirmed to have candidal blood-stream co-infection based on positive blood cultures.

Salient investigation results revealed hemoglobin values ranging from 9.1 to 14.8 gm/dl (mean: 12.6 gm/dl, normal range: 13.0-17.0 gm/dl), total leukocyte count ranging from 5,300 to 15,500/mm^3^ (mean: 10,600/mm^3^, normal range: 4,000-11,000/mm^3^), platelet values ranging from 96,000 to 600,000/mm^3^ (mean: 284,000/mm^3^, normal range: 150,000-400,000/mm^3^), D-dimer values ranging from 148 to 7,155 ng/ml (mean: 1,376 ng/ml, normal range: 0-243 ng/ml), and serum IL-6 values ranging from 14.6 to 6,337 pg/ml (mean: 714 pg/ml, normal range: 0-16 pg/ml). All patients had suggestive chest X-rays or HRCT chest findings consistent with COVID-19.

Except for one patient with a one-day history of bilateral visual loss, there were no complaints of recent visual loss or any other ocular symptoms among the remaining 24 patients. Pre-existing complaints included a single patient (4%, 56-year-old male) who notified us of poor vision in the right eye since childhood. Another patient (4%, 66-year-old male) provided a history of previous ocular treatment in the form of laser treatment with intravitreal injection in his left eye. A preliminary examination revealed no evidence of congestion or conjunctivitis that would preclude further examination.

A total of 50 fundus photographs were initially assessed for image quality. No media opacities were present that compromised retinal assessment. In 48 photographs, we found no evidence of fundus lesions. The presence of vascular disease (hemorrhages, occlusions), cotton wool spots, retinal opacification, or any areas of inflammation was specifically looked for. In the images of the two patients described above, we noted extensive chorioretinal atrophy consistent with myopic macular degeneration (56-year-old male) in his right eye and multiple discrete areas of chorioretinal atrophy consistent with photocoagulation for diabetic retinopathy in the left eye of the second patient (66-year-old male). These findings are summarized in Table 1.

**Table 1:** Summary of ocular, investigational, and important systemic features Hb: hemoglobin; TLC: total leukocyte count; DM: diabetes mellitus; HTN: hypertension; CKD: chronic kidney disease; ARDS: acute respiratory distress syndrome; MHD: maintenance hemodialysis; NIV: non-invasive ventilation; TIA: transient ischemic attack; IHD: ischemic heart disease; STEMI: ST-elevated myocardial infarction; NA: not available; ND: not done

Sr. no	RE fundus	LE fundus	Age (years)	Sex	Hb, gm/dl	TLC, k/mm^3^	Platelets, k/mm^3^	D-dimer, ng/ml	Chest imaging	Important systemic findings/outcomes
1	Normal	Normal	67	F	12.6	7.97	600	ND	Positive	NIV+ Intubation; died
2	Normal	Normal	39	M	14.5	5.69	283	480	Positive	NIV
3	Normal	Normal	40	M	NA	NA	NA	ND	Normal	Observation only
4	Normal	Normal	31	M	13.2	6.35	219	ND	ND	NIV only
5	Normal	Normal	57	M	10.1	5.3	96	641	Positive	CKD; HTN; DM; IHD; died
6	Normal	Normal	68	M	NA	NA	NA	ND	Positive	
7	Normal	Normal	60	M	12.6	18.5	479	605	Normal	DM; HTN; acute myocardial infarction (anterior wall STEMI)
8	Normal	Normal	31	M	15.8	11.35	316	148	Positive	NIV only
9	Normal	Normal	53	M	13.9	7.96	270	230	Positive	ARDS; died
10	Myopic degeneration	Normal	56	M	14.7	10.88	253	425	Positive	DM
11	Normal	Normal	58	F	11.5	15.52	179	7,155	Positive	Acute kidney injury; died
12	Normal	Normal	78	M	10.7	10.47	147	238	Positive	DM; HTN; IHD; intubation; died
13	Normal	Post-laser (macula)	66	M	9.1	4.81	258	4,352	Positive	DM; HTN; CKD; MHD; NIV+
14	Normal	Normal	79	M	10.7	7.9	300	ND	Positive	HTN; CKD; IHD; LVF
15	Normal	Normal	54	M	13.4	10.67	345	226	Positive	R frontal, parietal, and occipital infarcts
16	Normal	Normal	67	M	12	12.43	310	ND	ND	DM; IHD; CKD
17	Normal	Normal	60	M	13.6	15.51	349	ND	Positive	NIV
18	Normal	Normal	51	F	9.8	12.26	276	ND	Positive	Long segment thrombosis; right radial artery; NIV+
19	Normal	Normal	69	M	14.8	10.25	172	627	Positive	ARDS
20	Normal	Normal	43	F	10.4	13.25	354	2,770	ND	Distal popliteal artery thrombosis; left popliteal artery thrombectomy
21	Normal	Normal	48	M	15.5	13.2	370	461	Positive	Acute visual loss BE; CT brain normal (TIA)
22	Normal	Normal	35	M	NA	NA	NA	ND	Positive	Observation only
23	Normal	Normal	56	F	12.3	11	207	473	Positive	Candida co-infection; ARDS
24	Normal	Normal	46	M	13.5	12.28	200	1,822	Positive	Bilateral upper limb thrombus; died
25	Normal	Normal	60	M	NA	NA	NA	ND	Positive	Observation only

## Discussion

COVID-19 infection has a wide range of manifestations ranging from asymptomatic infections to more severe systemic disease, some of which require hospitalization or specialized intensive care treatment. Numerous risk factors have been reported related to the condition, including the presence of diabetes mellitus and concurrent cardiac or renal disease. SARS-CoV-2 infection is a microdroplet infection acquired via the respiratory route. The virus targets the cells of the bronchial epithelium that express angiotensin-converting enzyme 2 (ACE2), which results in a reduction of ACE2 expression with consequent lung injury. Subsequently, there is a widespread viremia with local infection as well as systemic disease as ACE2 cells are present in a wide range of tissues [4].

Theoretically, several mechanisms exist that may induce visual morbidity or detectable fundus lesions. These can include a systemic inflammation response syndrome (SIRS) that is common in severe systemic viral or bacterial infection and may affect the posterior segment. Additionally, infections of the retina or choroid can also occur either due to a direct viral infection as often seen with herpes simplex virus (HSV) or varicella-zoster virus (VZV) or due to the effect of co-infections that are common in patients with COVID-19. These include fungi such as *Candida albicans*, *Aspergillus flavus*, and *Aspergillus fumigatus*, or bacteria such as *Mycoplasma pneumoniae*, *Haemophilus influenzae*, and *Pseudomonas aeruginosa* [5].

A hallmark of SARS-CoV-2 infection is the presence of a concurrent prothrombotic state. Derangement of the coagulation pathway and utilization of clotting factors also occur, which may result in microthrombus formation and lead to a prothrombotic state and complications such as deep venous thrombosis, pulmonary embolism, strokes, or myocardial infarction. A prothrombotic state such as this could theoretically result in choroidal/retinal lesions such as vascular occlusions, choroidal infarctions, or hemorrhages [6]. A recent review by Abou-Ismail et al. suggests that venous thromboembolic events occur in 25-69% of patients, largely as pulmonary emboli, and arterial thromboembolic events occur in 3.7-16.3% of patients either as strokes and myocardial or limb ischemia [7].

Related coronavirus infections have caused pandemics in the recent past and these include the Middle East respiratory syndrome (MERS) and severe acute respiratory syndrome (SARS). There have been no reports of intraocular disease in either of these outbreaks, even though Loon et al. were able to isolate the SARS virus from the tears of three of 36 patients (8.3%) using PCR [8].

The initial reports of ocular involvement in SARS-CoV-2 described viral conjunctivitis. Guan et al. have described nine cases among a cohort of 1,099 patients in China [9]. Sindhuja et al. screened 127 patients with COVID-19 in Delhi, India, and of those, 12 patients (9.45%) were symptomatic and eight patients (6.29%) had conjunctival congestion consistent with conjunctivitis [10]. Kumar et al. have demonstrated the presence of SARS-CoV from conjunctival swabs in one of 45 patients (2.23%) using RT-PCR, suggesting a low level of viral detection [11]. Wu et al. screened 38 patients, of whom 12 had conjunctivitis but were able to detect SARS-CoV in only two (16.7%) among them [12].

The initial posterior segment findings were reported by Marinho et al. who described retinal and optical coherence tomography (OCT) findings in 12 patients. They demonstrated hyperreflectivity in the ganglion cell and inner plexiform layers in all 12 patients. Cotton wool spots and hemorrhages were seen in four patients [13]. Collison and Carroll have argued that these OCT findings represent normal anatomy rather than disease [14]. Invernezzi et al. evaluated 54 patients and detected hemorrhages in five patients (9.25%), cotton wools spots in four patients (7.4%), and dilated veins in 15 patients (27.7%). They noted that the mean arterial diameter and mean venous diameter were larger in COVID-19 patients [15]. The pathobiology of these cotton wool spots and hemorrhages is not clearly identified. The possibilities include direct viral cytopathic action and the incidental findings of diabetes mellitus or hypertension, which are common in these patients or as part of a non-specific SIRS. Casagrande et al. were able to detect SARS-CoV in the human retina in three of 14 eyes in an autopsy study [16].

Our findings are in agreement with Pirraglia et al. who studied a cohort of 43 admitted patients from Italy and noted no retinal lesions. The only positive finding was chorioretinitis in one eye, which was likely due to a co-infection [17]. They suggested a potential role of fundus examination in patients with co-infection. In contrast, Pereira et al. have reported the findings of a cohort of 18 hospitalized patients from Brazil, of whom 10 (55.5%) had detectable retinal lesions (hemorrhages, cotton wool spots) on dilated fundus examination via photography [18].

In this small series, we were not able to detect any fundus lesions. The limitations of our study include a patient population that largely comprised of patients with moderate to severe disease, thus creating a potential selection bias. Moreover, we were unable to assess the periphery, which was due to the difficulty in performing indirect ophthalmoscopy with protective face wear in this study, the resolution limits of the fundus camera, and a concern for the potential risk of non-detection of lesions.

We were also unable to do a comprehensive visual assessment or slit-lamp examination as the majority of the patients needed NIV/HFNO/ventilatory support and could not be shifted to the outpatient department. Additionally, the hospital COVID infection control committee did not permit any inter-departmental procedures so as to maintain a distinction between COVID-19 and non-COVID-19 zones.

## Conclusions

The initial reports of the ocular involvement in COVID-19 have described a primarily anterior segment disease in the form of ocular congestion and conjunctivitis. However, limited data is available on posterior segment findings. These lesions include cotton wool spots, hemorrhages, and vascular dilatation (both arterial and venous).

Our analysis of fundus photographs in this study revealed no evidence of vascular, inflammatory, or thromboembolic disease. A role may exist for the routine use of fundus examination in the characterization of COVID-19 infection, detection, and diagnosis of viral or bacterial co-infections or complications of prothrombotic states, but studies involving a larger cohort are required to confirm this. This would also enable the assessment of disease patterns in India as compared to other global centers and to determine whether ocular lesions can be correlated with systemic phenotypes and outcomes for all locations. The widespread use of OCT/OCT angiography may assist in the detection of subclinical disease.
